# Enzyme annotation in UniProtKB using Rhea

**DOI:** 10.1093/bioinformatics/btz817

**Published:** 2019-11-05

**Authors:** Anne Morgat, Thierry Lombardot, Elisabeth Coudert, Kristian Axelsen, Teresa Batista Neto, Sebastien Gehant, Parit Bansal, Jerven Bolleman, Elisabeth Gasteiger, Edouard de Castro, Delphine Baratin, Monica Pozzato, Ioannis Xenarios, Sylvain Poux, Nicole Redaschi, Alan Bridge, The UniProt Consortium

**Affiliations:** 1 Swiss-Prot Group, SIB Swiss Institute of Bioinformatics, Geneva 1211-4, Switzerland; 2 University of Lausanne, Lausanne 1015, Switzerland; 3 European Bioinformatics Institute (EMBL-EBI), Wellcome Genome Campus, Cambridge CB10 1SD, UK; 4 Protein Information Resource, Georgetown University Medical Center, Washington, DC 20007, USA; 5 Protein Information Resource, University of Delaware, Newark, DE 19711, USA

## Abstract

**Motivation:**

To provide high quality computationally tractable enzyme annotation in UniProtKB using Rhea, a comprehensive expert-curated knowledgebase of biochemical reactions which describes reaction participants using the ChEBI (Chemical Entities of Biological Interest) ontology.

**Results:**

We replaced existing textual descriptions of biochemical reactions in UniProtKB with their equivalents from Rhea, which is now the standard for annotation of enzymatic reactions in UniProtKB. We developed improved search and query facilities for the UniProt website, REST API and SPARQL endpoint that leverage the chemical structure data, nomenclature and classification that Rhea and ChEBI provide.

**Availability and implementation:**

UniProtKB at https://www.uniprot.org; UniProt REST API at https://www.uniprot.org/help/api; UniProt SPARQL endpoint at https://sparql.uniprot.org/; Rhea at https://www.rhea-db.org.

## 1 Introduction

The UniProt Knowledgebase (UniProtKB, at https://www.uniprot.org) is a reference resource of protein sequences and functional annotation that covers proteins from all branches of the tree of life (The UniProt Consortium, 2019). UniProtKB includes an expert curated core of over 560 000 reviewed UniProtKB/Swiss-Prot protein sequence entries that is supplemented by over 180 million unreviewed UniProtKB/TrEMBL entries annotated by automatic systems (release 2019_09 of October 16 2019). UniProtKB/Swiss-Prot curation focuses on experimentally characterized proteins from a broad range of taxa, including proteins of human origin (Breuza *et al.*, 2016) as well as proteins of bacteria, archaea, viruses and plants.

Approximately half of all protein sequence entries in UniProtKB/Swiss-Prot describe enzymes, whose function has traditionally been annotated using reference vocabularies such as the hierarchical enzyme classification of the Enzyme Nomenclature Committee of the IUBMB (often referred to as EC numbers) (Bairoch, 2000; McDonald *et al.*, 2009; McDonald and Tipton, 2014). In this article, we describe the introduction of a new reference vocabulary for the annotation of enzymes in UniProtKB—the Rhea knowledgebase of biochemical reactions (https://www.rhea-db.org) (Lombardot *et al.*, 2019; Morgat *et al.*, 2017). Rhea is a comprehensive expert-curated knowledgebase that uses the chemical ontology ChEBI (Chemical Entities of Biological Interest, https://www.ebi.ac.uk/chebi/) (Hastings *et al.*, 2016) to describe reaction participants, their chemical structures and chemical transformations. Rhea provides stable unique identifiers and computationally tractable descriptions for around 12 250 unique biochemical reactions, including reactions of the IUBMB enzyme classification, as well as thousands of additional enzymatic reactions, transport reactions and spontaneous reactions. The introduction of Rhea as the reference vocabulary for enzyme annotation will significantly improve the coverage and precision of enzyme annotation in UniProtKB. It will allow UniProtKB users to leverage knowledge of chemical structures for a wide variety of applications including the integrative analysis of metabolomics and other ‘omics data (Kale *et al.*, 2016; Sud *et al.*, 2016), the study of enzyme chemistry and evolution (Rahman *et al.*, 2016; Tyzack *et al.*, 2019), the construction and annotation of metabolic models (Cottret *et al.*, 2018; King *et al.*, 2016; Moretti *et al.*, 2016) and the engineering of pathways for biosynthesis and bioremediation (Duigou *et al.*, 2019).

In the following we describe the annotation of enzyme data in UniProtKB using Rhea as well as modifications to the UniProt website, API and SPARQL endpoint that allow UniProt users to exploit this enhanced enzyme dataset.

## 2 Materials and methods

### 2.1 Rhea as a reference vocabulary for enzyme annotation in UniProtKB

Prior to this work, UniProtKB used the hierarchical enzyme classification of the Enzyme Nomenclature Committee of the IUBMB (hereafter referred to as the IUBMB Enzyme Classification) as the main reference vocabulary for enzyme annotation. The IUBMB Enzyme Classification uses a hierarchy of exactly four levels to classify enzymes according to the chemistry of representative reactions (Bairoch, 2000; McDonald *et al.*, 2009; McDonald and Tipton, 2014). In this work we introduce Rhea reaction identifiers as the reference vocabulary for enzyme annotation in UniProtKB, with the corresponding four digit enzyme class (EC number) now selected from a distinct mapping of Rhea reactions to EC numbers (this mapping is maintained by Rhea at https://www.rhea-db.org/download). Note that Rhea contains thousands of reactions that are not described by the IUBMB Enzyme Classification—reactions that have no corresponding EC number—and so EC number annotations for Rhea reactions are optional in UniProtKB. Note also that the IUBMB Enzyme Classification may describe enzymatic reactions whose specific chemistry is not yet well characterized and which cannot be described using ChEBI. These reactions do not appear in Rhea and we continue to describe them in UniProtKB in text form. In summary, there are now three main options for describing enzymatic reactions in UniProtKB:

– using Rhea reaction identifiers that map to EC numbers;
– using Rhea reaction identifiers that do not map to EC numbers;
– using textual descriptions that map to EC numbers.

### 2.2 Migration of legacy enzyme annotation in UniProtKB to Rhea and re-annotation

In order to lay the groundwork for the integration of Rhea in UniProtKB, we first mapped legacy textual descriptions of enzymatic reactions in UniProtKB to Rhea reaction identifiers. We accomplished this using the ENZYME database (Bairoch, 2000), which links these textual descriptions of reactions to their corresponding EC numbers, and the Rhea database, which links EC numbers to their corresponding Rhea reactions. We checked and validated all such mappings of [UniProtKB enzyme annotation]—[EC number]—[Rhea identifier] derived in this way. A small number of legacy UniProtKB enzyme annotations were not based on EC numbers, and we mapped these textual descriptions manually to Rhea identifiers where possible, creating new Rhea reactions where needed. We then used the completed mapping to replace the legacy textual descriptions of enzymatic reactions in UniProtKB by Rhea annotations, and to update all automatic annotation rules that are used to add enzyme annotations to UniProtKB/TrEMBL records, including those from HAMAP (Pedruzzi *et al.*, 2015) and PROSITE (Sigrist *et al.*, 2013). Mapping of all EC number annotations is now complete, while the mapping of additional legacy enzyme data described in natural language in other annotation comments in UniProtKB/Swiss-Prot (mainly in ‘FUNCTION’ annotation comments) is still ongoing.

### 2.3 UniProt tools and services that use Rhea

We modified the UniProt data model and output formats—text, XML and RDF—to include Rhea reaction data and references to ChEBI. We modified the UniProt website https://www.uniprot.org (Jain *et al.*, 2009), UniProt REST API https://www.uniprot.org/help/api and SPARQL endpoint https://sparql.uniprot.org/ to support searches using Rhea and ChEBI identifiers as well as ChEBI names, synonyms and chemical structures represented as InChIKeys. The InChIKey, a simple hash representation of chemical structures, provides a convenient means to search and map chemical structure databases. It encodes information on connectivity, stereochemistry and charge in three distinct ‘layers’. A more complete description of the InChIKey is available at https://www.inchi-trust.org/.

## 3 Results

### 3.1 Annotation of UniProtKB using Rhea

We performed a complete re-annotation of legacy UniProtKB enzyme data using Rhea (as described in Methods), and now use Rhea as the primary reference vocabulary for enzyme annotation in UniProtKB. UniProtKB/Swiss-Prot currently includes annotations for 6654 unique Rhea reactions (around 54% of all Rhea reactions), which feature in 216 785 distinct UniProtKB/Swiss-Prot protein records (38.6% of all UniProtKB/Swiss-Prot records are annotated with Rhea) (release 2019_09 of October 16 2019). Of the 6654 Rhea reactions used in UniProtKB/Swiss-Prot, 4938 reactions (around 75%) are linked to EC numbers. We are currently working to improve the coverage of the 5593 Rhea reactions not currently represented in UniProtKB/Swiss-Prot through a variety of approaches. These approaches include continuing expert curation of new literature, re-annotation of free text legacy annotations in UniProtKB/Swiss-Prot entries, and integration of data from other resources that use Rhea. One such resource of note is the SwissLipids knowledgebase (Aimo *et al.*, 2015), which contains annotations for more than 1400 unique Rhea reactions that are not yet represented in UniProtKB. We will describe these and other annotation efforts in more detail in forthcoming publications.

### 3.2 UniProt tools and services that use Rhea

Below we describe how users can navigate and exploit Rhea data using the UniProt website, REST API and SPARQL endpoint.

#### 3.2.1 Rhea and the UniProt website

**Fig. 1.**
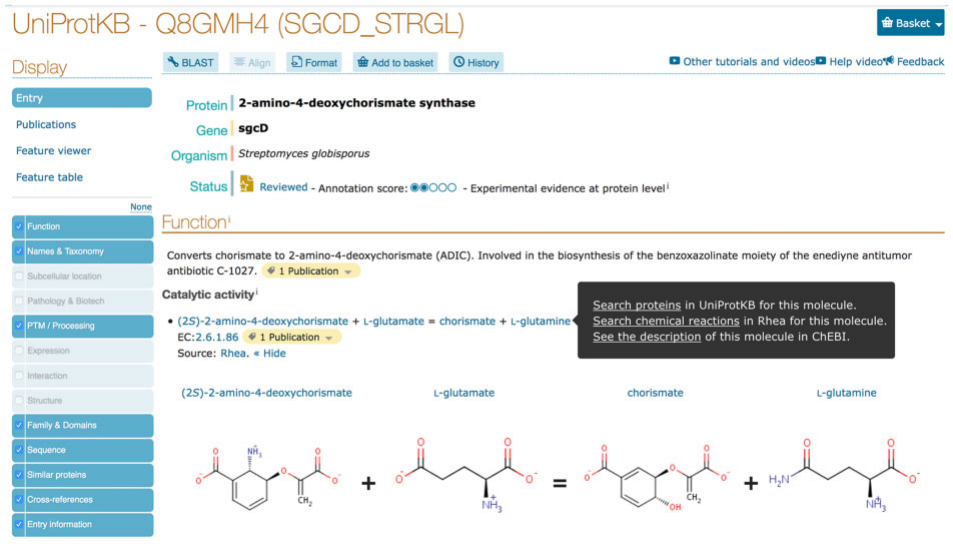
UniProtKB entry view showing Rhea annotation for the *Streptomyces globisporus* enzyme 2-amino-4-deoxychorismate synthase (UniProt: Q8GMH4 annotated with RHEA: 25512). The search and link-out options available for each reaction participant are illustrated using ‘l-glutamine’; users can search in UniProtKB or Rhea, or link out to ChEBI to learn more about the metabolite in question. We omit most sections for clarity

The UniProt website https://www.uniprot.org constitutes the main point of entry for most UniProt users. The UniProtKB entry view provides a summary of annotated Rhea reactions for each enzyme (Fig. 1). Users can choose to reveal the two-dimensional structures of reaction participants for each annotated reaction, as well as click on the reactions and their participants to launch searches in UniProtKB or Rhea or link out to ChEBI.

**Fig. 2.**
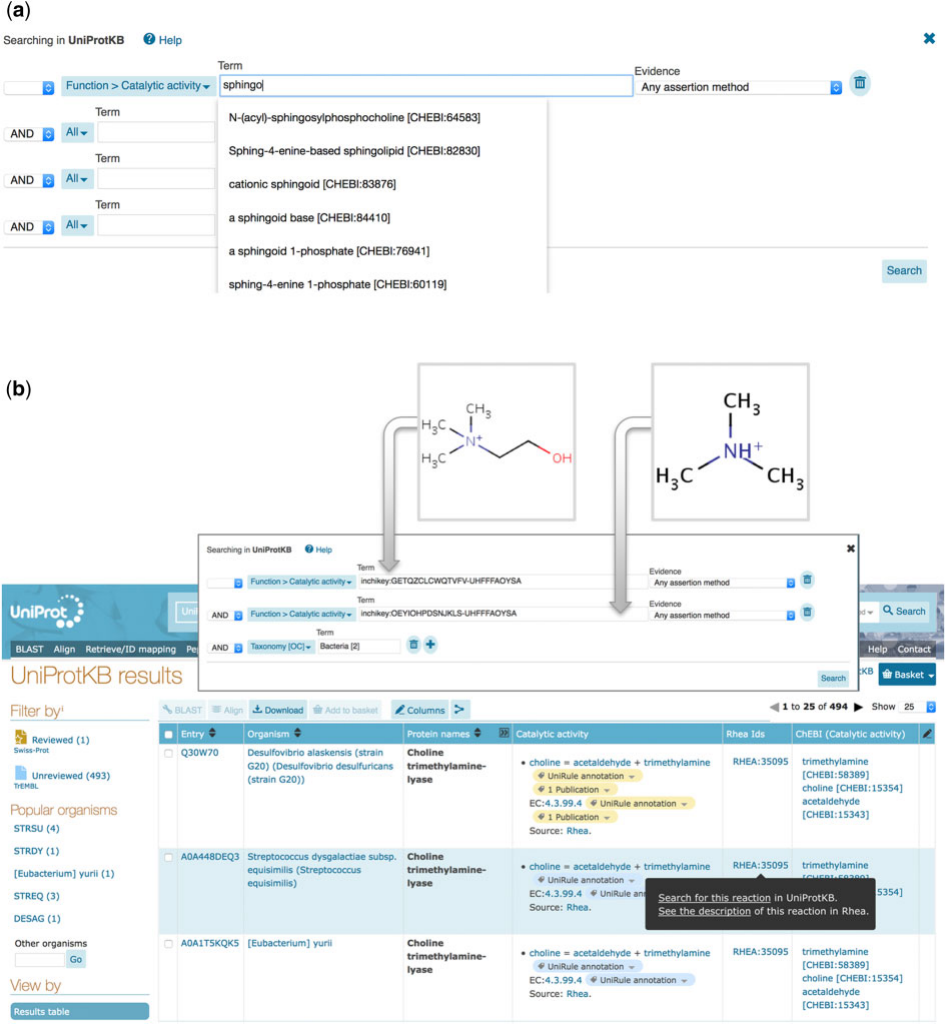
Advanced search in UniProtKB for enzymatic reactions. (**a**) Advanced search using chemical nomenclature. The autocomplete feature is shown. (**b**) Advanced search using InChIKeys of choline (HMDB00097) and trimethylamine (TMA) (HMDB0000906) to identify bacterial enzymes metabolizing both compounds. The result table can be customized to display Rhea reaction data, which can be used to launch further searches and link out precisely as in the entry view

Figure 2 illustrates selected advanced search options for reactions, chemical names and structures in UniProtKB. Users can search for identifiers from Rhea as well as identifiers, names, synonyms (Fig. 2a) and chemical structures (encoded as InChIKeys) from ChEBI (Fig. 2b). The complete ChEBI ontology is indexed to support hierarchical searches, while ChEBI identifiers entered by users are mapped to those of the major species at pH 7.3, the form used in Rhea, using the mapping provided at https://www.rhea-db.org/download.

InChIKeys provide a means to query UniProtKB using chemical structure data from other resources, including reference knowledgebases such as the Human Metabolome Database (HMDB) (Wishart *et al.*, 2018) or LIPID MAPS (Fahy *et al.*, 2009). Users of these and other resources can simply convert their structures to InChIKeys and use them to query UniProtKB. Searches may be performed using the complete InChIKey (to find exact structure matches), or using the first and second layers of the InChIKey (to find molecules with matching connectivity and stereochemical orientation, irrespective of charge, as in Fig. 2b), or using only the first layer of the InChIKey (to find molecules with matching connectivity).

Figure 2b illustrates an InChIKey search for microbial enzymes that metabolize choline (HMDB00097, InChIKey=OEYIOHPDSNJKLS-UHFFFAOYSA-N) and trimethylamine (TMA) (HMDB0000906, InChIKey=GETQZCLCWQTVFV-UHFFFAOYSA-N). TMA produced by the gut microbiome can have a profound impact on the health of the human host. Gut microbes convert dietary choline to TMA, which is subsequently absorbed and converted to the pro-atherogenic molecule trimethylamine N-oxide (TMAO) (HMDB0000925) by enzymes of the human host liver such as FMO3 (Canyelles *et al.*, 2018; Chhibber-Goel *et al.*, 2017). This InChIKey search allows users of HMDB (and other resources) to exploit UniProtKB to connect circulating metabolites from the microbiome such as TMA to the enzymes that produce them. In this case, that is homologs of the choline trimethylamine-lyase cutC of *Desulfovibrio alaskensis* (UniProtKB: Q30W70).

For those chemical structures that have no corresponding match annotated in UniProtKB—no matching InChIKey first (connectivity) layer—users might choose instead to search for relevant information about the chemical classes to which these structures belong. They might select relevant chemical classes themselves from the ChEBI ontology, using their own expert knowledge of the ChEBI classification, or might choose to map chemical structures of interest to their corresponding ChEBI classes using computational tools such as ClassyFire (Djoumbou Feunang *et al.*, 2016). Interested readers can find further information about chemical data search in UniProtKB in the online documentation provided (see https://www.uniprot.org/help/chemical_data_search).

#### 3.2.2 Rhea and the UniProt REST API

The UniProt website serves a REST API (https://www.uniprot.org/help/api) that allows users to query and process data programmatically. The REST API has been modified to handle Rhea and ChEBI identifiers, as well as ChEBI names, synonyms and structural data. The sample REST API query shown below recapitulates the UniProt website query shown in Figure 2b, and will retrieve bacterial enzymes that metabolize choline and trimethylamine using their respective InChIKeys.

annotation:(type:"catalytic activity" inchikey:OEYIOHPDSNJKLS-UHFFFAOYSA) annotation:(type:"catalytic activity" inchikey:GETQZCLCWQTVFV-UHFFFAOYSA) taxonomy:"Bacteria [2]"

As before, this particular query uses only the first and second layers of the InChIKey to allow permissive matching between different charge state representations. The query is available at https://tinyurl.com/y2mcjotd. Users can modify REST queries like that shown above in order to specify both the required data output (annotation fields) and format (such as .tab, .xls, .rdf and others) (for more details see https://www.uniprot.org/help/api_queries).

#### 3.2.3 Rhea and the UniProt SPARQL endpoint

**Fig. 3.**
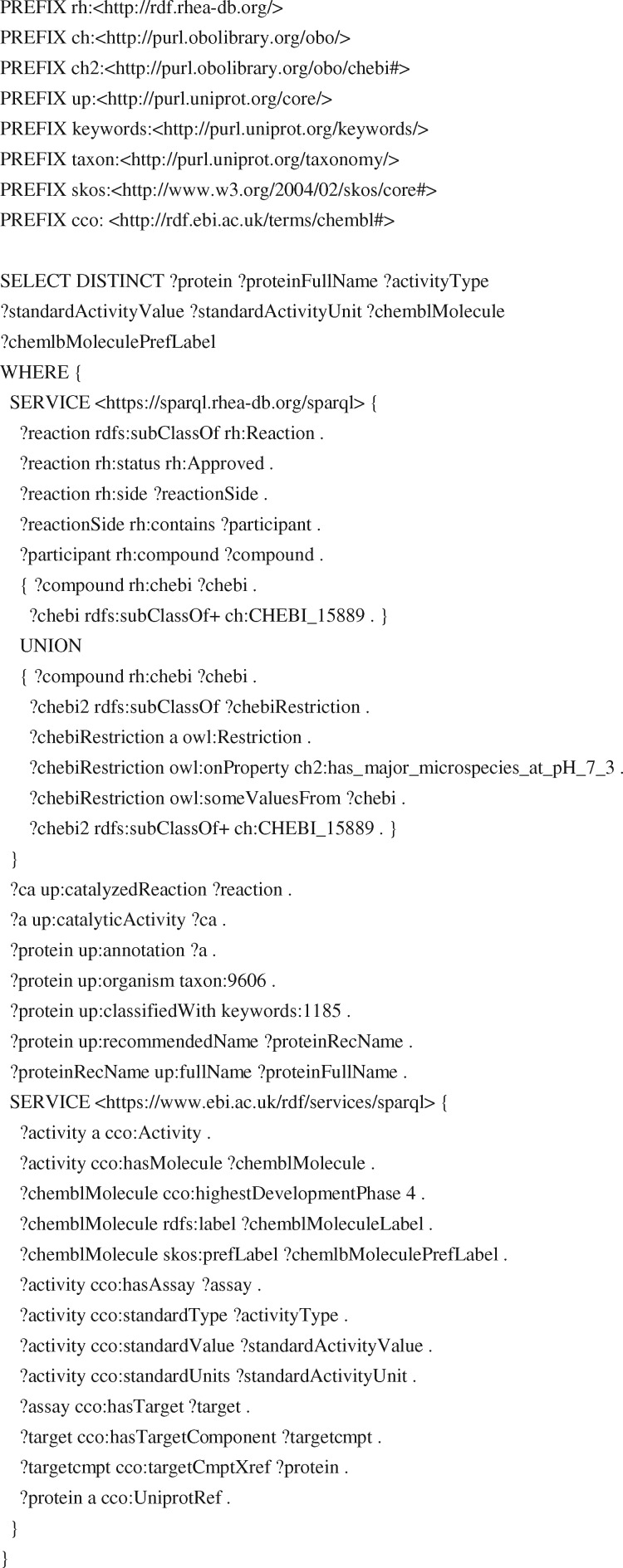
A sample federated SPARQL query that leverages Rhea annotation in UniProtKB. The query retrieves information about drugs that target enzymes involved in human sterol (ChEBI: 15889) metabolism from the UniProt, Rhea and ChEMBL SPARQL endpoints, federating the three SPARQL endpoints with two SERVICE calls

The UniProt SPARQL endpoint https://sparql.uniprot.org/ allows users to perform complex federated queries that combine RDF data from UniProt with that from other SPARQL endpoints. Like the UniProt website and REST API, the UniProt SPARQL endpoint now supports queries using Rhea and ChEBI identifiers, as well as ChEBI names, synonyms and structural data. Figure 3 provides a sample SPARQL query that combines the UniProt, Rhea (Lombardot *et al.*, 2019) and ChEMBL (Gaulton *et al.*, 2017) SPARQL endpoints. This query exploits the ChEBI ontology to retrieve those drugs that target human enzymes acting on cholesterol or other sterols (members of the ChEBI class ChEBI: 15889). The aforementioned pro-atherogenic metabolite TMAO perturbs cholesterol and sterol metabolism (Canyelles *et al.*, 2018; Chhibber-Goel *et al.*, 2017), and drugs that target these processes might be useful to investigate or even modulate these effects.

The query shown in Figure 3 makes use of the ChEBI ontology to find information relevant to cholesterol and other sterols. Users might also identify derivatives of cholesterol or cholesterol like molecules using SPARQL endpoints that support chemical similarity or chemical substructure searches over ChEBI, such as the Integrated Database of Small Molecules (IDSM) (Kratochvil *et al.*, 2019). These advanced search capabilities could be further combined with those of a range of other SPARQL endpoints from resources such as Ensembl (Zerbino *et al.*, 2018), OMA (Altenhoff *et al.*, 2018), OrthoDB (Kriventseva *et al.*, 2019) and Bgee (Bastian *et al.*, 2008), in order to explore small molecule metabolism in the context of genomic organization, variation, evolution and anatomy. We describe more sample queries in the documentation available at the UniProt SPARQL endpoint.

## 4 Conclusions and future directions

Here we describe the introduction of Rhea as the reference vocabulary for enzyme annotation in UniProtKB as well as a range of tools and services that allow UniProt users to exploit this enhanced enzyme dataset. Future work will focus on a number of areas. First, we will continue to expand the coverage of Rhea in UniProtKB through expert curation—for human metabolism as well as that of microbes, plants and fungi, including both primary and secondary metabolism. Second, we will extend the use of ChEBI to describe all small molecule chemical structure data in UniProtKB, including functionally important ligands and post-translational modifications (see https://www.uniprot.org/docs/ptmlist). Third, we will continue to develop more sophisticated—and user-friendly—chemical and biological search capabilities for UniProtKB that extend those described here.

The ongoing standardization of small molecule data in UniProtKB using Rhea and ChEBI will provide a basis to improve links and interoperability with other biological knowledge resources that also use these reference vocabularies (and the chemistry standards that they employ). Other users of Rhea include the Gene Ontology (The Gene Ontology Consortium, 2019) and Reactome (Fabregat *et al.*, 2018), which recently adopted Rhea as the reference for enzymatic reaction chemistry (Chris Mungall and Peter D’Eustachio, personal communication), and the open chemistry database PubChem (Kim *et al.*, 2019). Other users of ChEBI include the metabolomics data repository MetaboLights (Kale *et al.*, 2016), the IMEx molecular interaction databases (Orchard *et al.*, 2012) and the Complex Portal (Meldal *et al.*, 2015), the literature annotation services of Europe PubMed Central (Europe PMC Consortium, 2015), the BioModels repository (Glont *et al.*, 2018) and the Immune Epitope Database (IEDB) (Dhanda *et al.*, 2019). Each of these resources can now leverage UniProtKB as a source of additional biological information for small molecule data, as might any resource that uses the same standard chemical structure descriptors. We hope that these, and other users and resources, will find new ways to exploit the enhanced enzyme annotations in UniProtKB, and look forward to discovering them.
